# miR390 family of *Cymbidium goeringii* is involved in the development of reproductive organs in transgenic *Arabidopsis*

**DOI:** 10.1186/s12870-022-03539-3

**Published:** 2022-03-26

**Authors:** Zihan Xu, Qian Liu, Yue Chen, Yuanhao He, Fengrong Hu

**Affiliations:** 1 College of Landscape Architecture, Nanjing Forestry University, 210037 Nanjing, Jiangsu Province China; 2 Institute of Horticulture, Zhejiang Academy of Agricultural Sciences, 310021 Hangzhou, Zhejiang Province China

**Keywords:** *Cymbidium goeringii*, miR390, Reproductive organ, Flower development, *Arabidopsis*

## Abstract

**Background:**

miR390s is an ancient family with a high level of conservation among plant miRNAs. Through the auxin signal transduction pathway, miR390 participates in diverse biological processes of plant growth and development. As an important Chinese traditional orchid, *Cymbidium goeringii* has unique flower shape and elegant fragrance. But its development has been greatly restricted because of the low flower bud differentiation and the difficult reproduction. This study aims to provide guidance for the role of cgo-miR390 in reproductive organ development to enhance the ornamental and economic value of *Cymbidium*.

**Results:**

*MIR390a*, *MIR390b* and *MIR390c* of *C. goeringii* were cloned, and their length ranged from 130 to 150 nt. Each precursor sequence of cgo-miR390 contains 2 to 3 mature miRNAs. Three kinds of cgo-miR390s displayed distinct temporal and spatial expression patterns during floral development in *C. goeringii*. The overexpression of *MIR390s* alters morphology and function of stamens and pistils in *Arabidopsis*, such as enlargement of anther aspect ratio and separation of stylar and stigmas, which affects the development of fruits and seeds. In particular, the pollen amount decreased and the seed abortion rate increased in *cgo-MIR390c*-overexpressed plants.

**Conclusions:**

cgo-miR390 family affected the development of reproductive organs in transgenic *Arabidopsis*. The study provides references for the genetic improvement for orchid with potentially great economic benefit.

**Supplementary Information:**

The online version contains supplementary material available at 10.1186/s12870-022-03539-3.

## Background

MicroRNAs (miRNAs) are a class of non-coding endogenous small RNAs with 20–24 nucleotide (nt) in length [1], which have been identified as new regulators of gene expression at transcriptional, post-transcriptional and post-translational levels [2]. The ancient and highly conserved miRNA, miR390, plays an important role in diverse processes of plant growth and development, including apical dominance, leaf senescence, root formation and abiotic and biotic stress responses, through the auxin signal transduction pathway. According to the miRbase database, miR390 family comprises no more than 4 members in most plants, such as only two members in *Arabidopsis thaliana* [3]. It has shown that some Auxin response factors (*ARFs*) are the target genes of miR390. Notably, miR390 triggers the production of trans-acting (ta) siRNAs from *TAS3* transcripts, which regulated *ARFs* in trans [4, 5].

miR390-*TAS3*-*ARF* regulatory network confers sensitivity and stability to auxin responses in various tissues and developmental stages. During root development, an auxin-response element (*AuxRE*) in the promoters of miR390 was bound by *ARF5*/*MONOPTEROS* (*MP*), which affects the expression of miR390 in the transit-amplifying compartment of the root meristem. miR390 then modulated the abundance of *ARF2*/*3*/*4* by *TAS3* [6]. miR390 was also specifically expressed at the sites of lateral root (LR) initiation where it triggers the biogenesis of ta-siRNAs to inhibit *ARF2*/*3*/*4*, and these ARFs affect auxin-mediated 390 accumulation as well [7, 8]. Therefore, an *ARF*-miR390-*TAS3* tasiRNA-*ARF* regulatory network was established due to the positive and negative feedback regulation of miR390 by *ARF2*/*3*/*4*. In leaf development, it is likely that AGO associates with miR390 and cleaves *TAS3* precursor RNAs as ta-siRNAs. After *DCL4*-mediated processing, this complex cleaves their targets, including *ARF3* and *ARF4*, to regulate leaf morphology development in *Arabidopsis* [9–11]. Dissimilarly, the expression of miR390 in leaf polarity of maize is established and maintained independently of the ta-siRNA pathway [12]. In addition, miR390a has been previously reported to be involved in dark-induced leaf senescence, while *ARF2*, a target gene of miR390, positively regulated auxin-mediated leaf longevity in *Arabidopsis* [13–15].

Research in the floral development of miR390 is more limited. It has been reported that *TAS3* ta-siRNA, whose primary transcripts were processed by miR390, extended the juvenile phase and delayed the flowering phase through negative regulation of *ARF3* [16, 17]. Moreover, the analysis of miRNAs and degradome in four stages of fruit development, including young fruit stage, fruit expansion stage, fruit coloring stage, and full maturity stage, found that *TAS3a* and *TAS3b* were targeted and furtherly degraded by miR390 in tomato. LemiR390 showed a high level and negatively regulated the expression level of *LeTAS3* during the early stage, especially 1–2 weeks after flowering [18].

Taken together, miR390 plays critical roles in the regulation of plant growth and development, which can be used as a promising object for breeding and genetic improvement in ornamental plants. Moreover, the effects of miR390 have been extensively studied in vegetative organs development, but less in reproductive organs. This is an innovative point in this study. As an important Chinese traditional orchid, *Cymbidium goeringii* has unique flower shape and elegant fragrance. But its development has been greatly restricted because of the low flower bud differentiation and the difficult reproduction. Here, we try to provide guidance for the role of a miRNA family in reproductive organ development. Recently, we obtained three predicted mature miR390s and their precursor sequences, designated cgo-miR390 family, from small RNA sequencing data in *C. goeringii*. This present study was aimed to investigate the expression patterns of miR390s in *C. goeringii* and their functional roles in *Arabidopsis*. The results suggest that cgo-miR390 family is a regulator involved in the development of reproductive organs in orchid plants.

## Results

### Sequence analysis of miR390s in *Cymbidium goeringii*

**Fig. 1:**
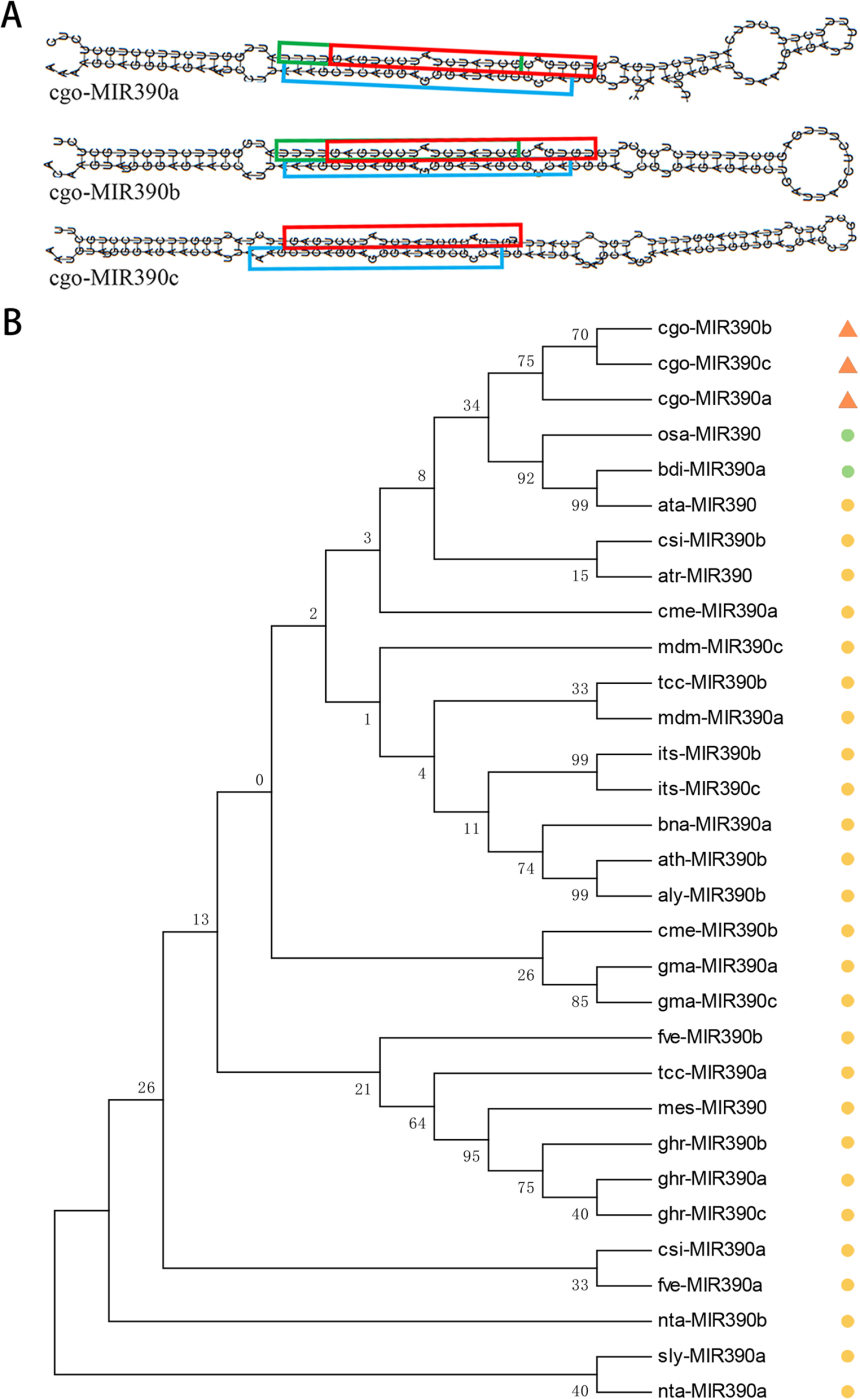
Sequence analysis of *cgo-MIR390s*. **A** The secondary structures of *cgo-MIR390s* in *Cymbidium goeringii*. **B** The phylogenetic relationship of *cgo-MIR390s* with other homologous sequences. The triangles represent *C. goeringii*, the green dots represent monocotyledons, and the yellow dots represent dicotyledons

*MIR390a*, *MIR390b* and *MIR390c* sequences were identified based on the transcriptome sequence data. Then the three sequences were cloned from the leaf of *C. goeringii* ‘Songmei’, and their length ranged from 130 to 150 nt (Table S1). Sequencing results showed that these three *cgo-MIR390s* produced a total of 8 mature miRNAs, in which the sequence of 3 mature miRNAs each detected on *MIR390a* and *MIR390b* were identical (Table 1; Fig. 1 A). Predicted stem-loop structures of three precursor sequences were shown in Fig. 1, all of them had 2 nt overhang structure at the distal end of their arms, which resulted from *Dicer* cleavage [19]. According to reference [20], the minimal folding free energy indexes (MFEIs) of *cgo-MIR390a*/*b*/*c* were 1.00, 1.35 and 1.18, respectively. These calculation results indicated that the sequences were all most likely to be miRNA due to the MFEIs were more than 0.85. In addition, no secondary stems or large loops interrupted the miRNA:miRNA* duplex, and there were fewer than 5 mismatched positions in duplex. These features are consistent with the universal characteristics of pre-miRNAs [21].

**Fig. 2:**
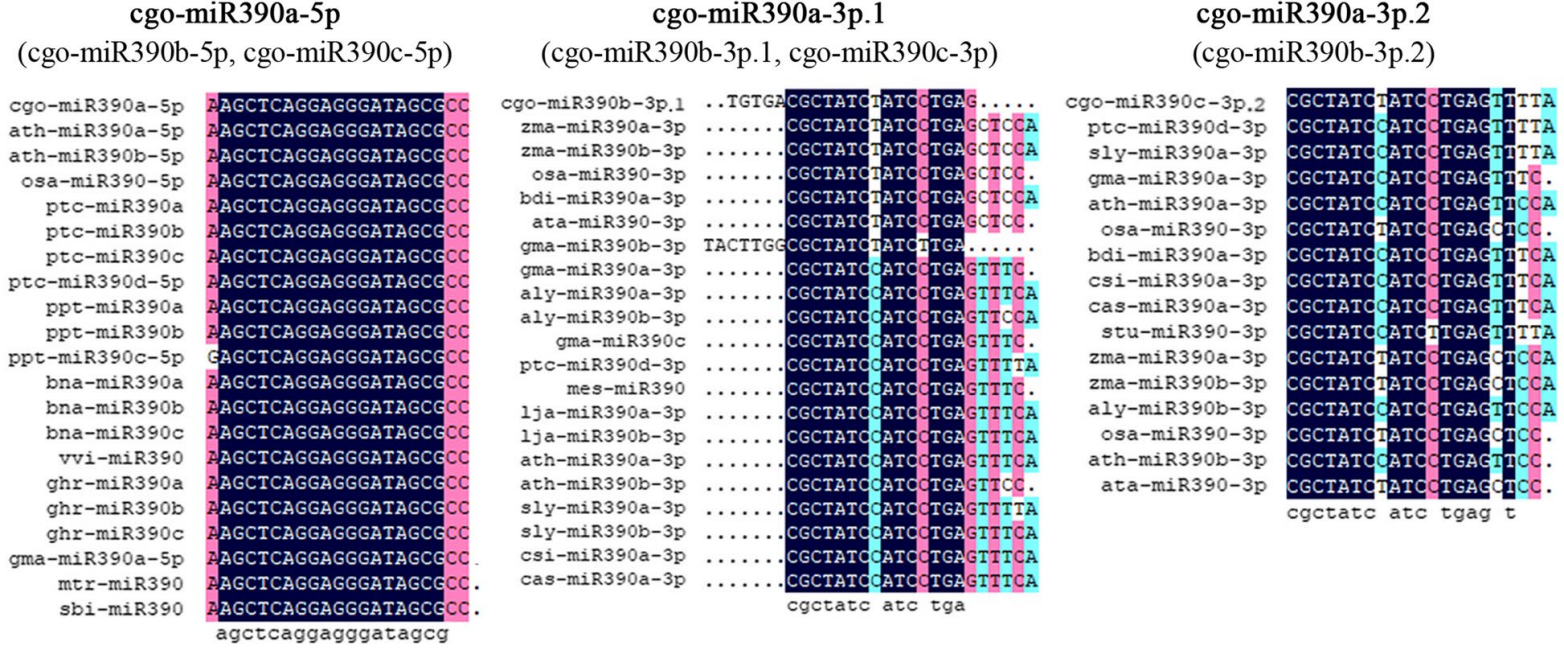
Nucleotide sequence alignment of cgo-miR390s with other homologous sequences

Analysis of homologous sequences found that three *MIR390* genes of *C. goeringii* showed strong homology, and all clustered together within the same branch of the phylogenetic tree (Fig. 1B). Furthermore, *MIR390s* in monocot plants display a higher sequence identity with *cgo-MIR390s* than those in dicots. It is obvious that this kind of pre-miRNA in monocot and dicot plants underwent evolutionary diversification. For analysis of mature miRNAs, cgo-miR390a-5p (cgo-miR390b-5p, cgo-miR390c-5p) was the most conserved one, followed by miR390a-3p.1 (miR390b-3p.1, miR390c-3p) and miR390a-3p.2 (miR390b-3p.2) (Fig. 2). This may be related to the evolution of miR390 family in *C. goeringii* as well.

**Table 1:** List of gene information

Gene type	Gene name	Inclusion relation	Sequence length
Precursor sequences	*cgo-MIR390a*	● ■ ▲	145bp
	*cgo-MIR390b*	● ■ ▲	133bp
	*cgo-MIR390c*	● ■	150bp
Mature miRNAs	cgo-miR390a-5p	●	21nt
	cgo-miR390b-5p	●	21nt
	cgo-miR390c-5p	●	21nt
	cgo-miR390a-3p.1	■	21nt
	cgo-miR390b-3p.1	■	21nt
	cgo-miR390c-3p	■	21nt
	cgo-miR390a-3p.2	▲	21nt
	cgo-miR390b-3p.2	▲	21nt

### Expression analysis of cgo-miR390s in *Cymbidium goeringii*

**Fig. 3:**
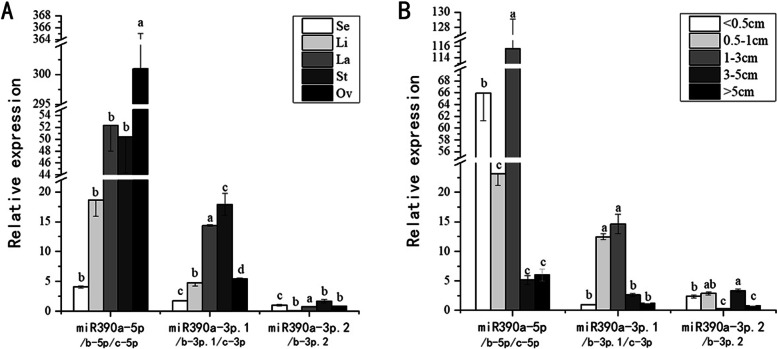
Expression patterns of cgo-miR390s in *Cymbidium goeringii*. **A** Expression level of miR390s in various floral organs. Se, sepal; Li, lip; La, lateral petal; St, stamen; Ov, ovary. **B** Expression level of miR390s in developing stages of floral buds. The mean lengths of flower buds at different stage were expressed in cm. The different letters indicate significant differences among 5 samples within a single group (*P* < 0.05)

All three miR390s were expressed in each floral organ of fully open flowers. The expression of cgo-miR390a-5p was specific to the ovary (Fig. 3 A), whereas cgo-miR390a-3p.1 was mainly expressed in lateral petal and stamen. By contrast, the expression of cgo-miR390a-3p.2 was extremely low in all floral organs. During flower bud development, cgo-miR390a-5p and cgo-miR390a-3p.1 had the highest expression when the flower bud length was 1-3 cm (Fig. 3B), while cgo-miR390a-3p.2 has the lowest at that time. When the length of flower buds was 3-5 cm, these three miR390s were all expressed at about the same level. These three kinds of cgo-miR390s showed overall non-overlapping expression patterns both temporally and spatially, which suggested they probably have functional divergence in regulating *Cymbidium* flower development (Fig. 3).

### Effect of cgo-miR390s on stamens and pistils development in *Arabidopsis*

**Fig. 4:**
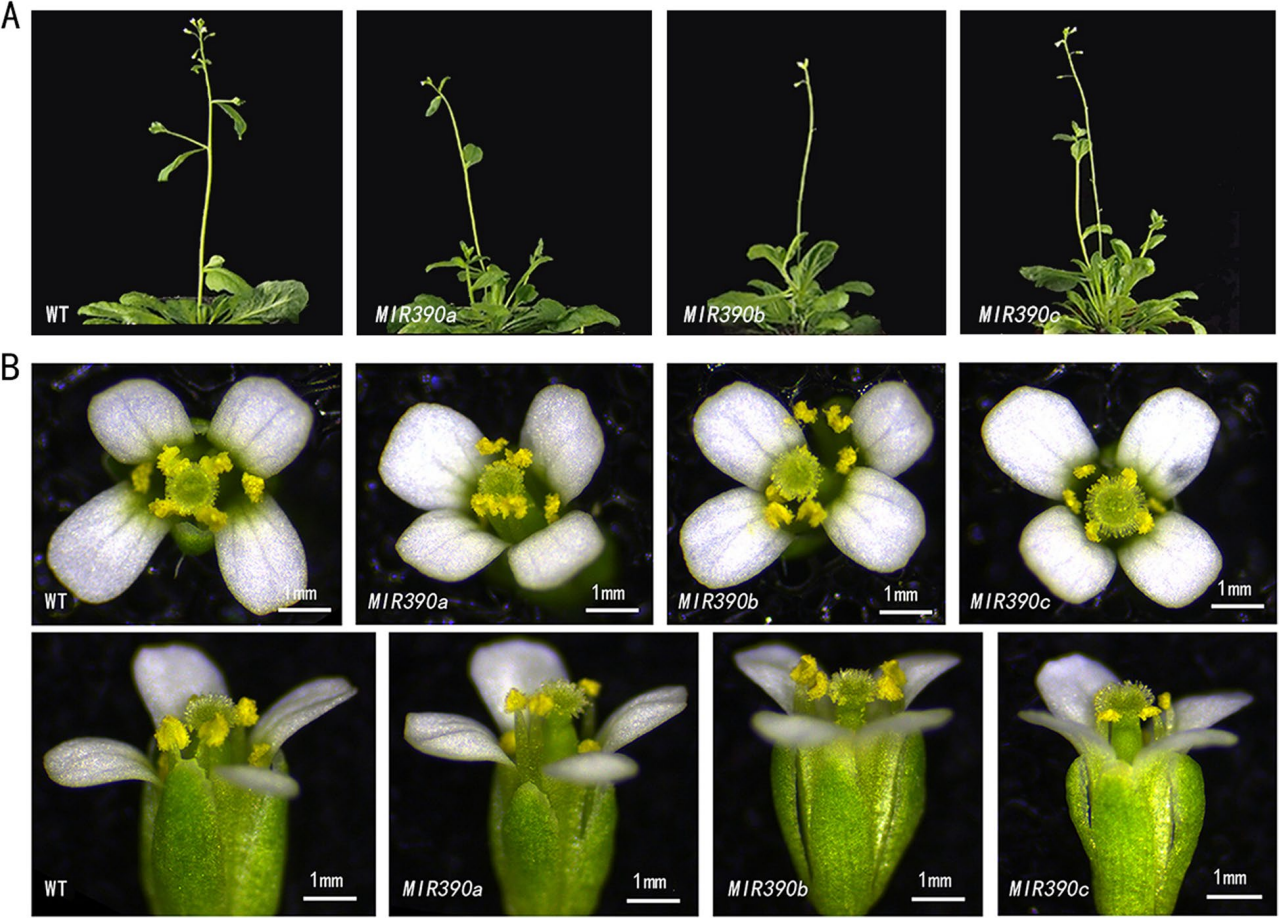
**A** Flowering time observation of wild type and transgenic *Arabidopsis* plants. **B** Flower characterization of wild type and transgenic *Arabidopsis* plants. The first line showed the top view of flowers and the second line showed the side view of flowers under a stereomicroscope

To investigate the function of cgo-miR390 family in flower development, we overexpressed *cgo-MIR390s* in *Arabidopsis*. The flowering time of wild-type and transgenic plants was almost consistent (Fig. 4A). By observing the external morphology of fully open flowers, compared with wild-type (WT) plants, the pollen attached to the anthers of the three transgenic plants was less, which was most obvious in *cgo-MIR390c*-overexpressing *Arabidopsis* (Fig. 4B). We speculate that this might be due to anthers’ early maturation, early dehiscence and insufficient pollen production.

**Fig. 5:**
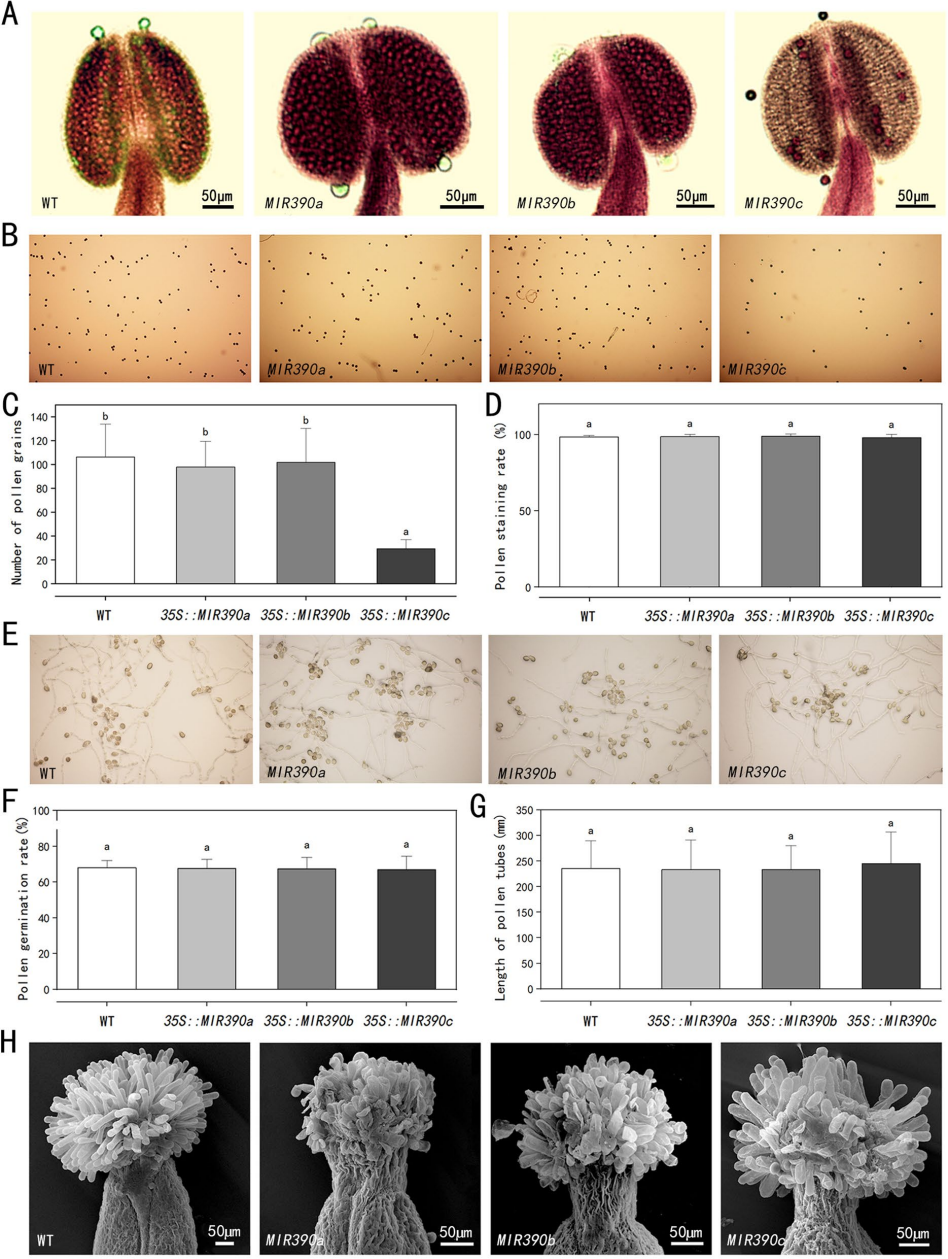
Phenotype changes in floral organs of wild type and transgenic *Arabidopsis* plants. **A** The stamens staining with Alexander solution under an optical microscope. **B** The pollen grains staining with Alexander solution under an optical microscope. **C**-**D** The statistical results of the pollen stains’ number and staining rate under an optical microscope with 10×magnification. **E** In vitro pollen germination and pollen-tube growth under an optical microscope with 20×magnification. **F**-**G** The statistical results of pollen germination rate and pollen tubes’ length. **H** The stigma of pistils under an electron microscope. The different letters indicate significant differences (*P* < 0.05)

Alexander staining was performed on the stamens of unopened flowers to further confirm this phenomenon. Figure 5 A showed that the anther aspect ratio (anther width/anther length) of three *cgo-MIR390s*-overexpressed plants was all larger than that of wild-type, suggesting that these three genes all regulated the morphological development of stamens. At this time, the pollen of wild-type did not reach full maturity. In contrast, pollen cells of *cgo-MIR390a* and *MIR390b* overexpressing plants were fully mature and kept inside the locules of anther, while the anther of *cgo-MIR390c*-overexpressing plants was prematurely dehisced and pollen grains were released (Fig. 5 A). Figure 5B-D also illustrated that the pollen amount from fully open flowers of *cgo-MIR390s*-overexpressed plants, especially *MIR390c*, was lower than the wild-type, but the pollen viability was generally as high as that of WT. Moreover, in vitro pollen germination assays showed normal pollen germination and pollen tube growth in transgenic plants (Fig. 5E-G).

The pistil morphologies also changed in transgenic *Arabidopsis*, mainly in the stigma. Figure 5 H showed that the stigma papilla cells of wild-type plants exhibited a finger-like shape and were tightly arranged, while the majority of papilla cells of *cgo-MIR390a* and *MIR390b* overexpressed plants are shrunken. This kind of cells of *MIR390c*-overexpressed plants displayed morphology similar to the wild-type but was more loosely arranged. In addition, all transgenic plants split styles and stigmas. These changes in stamens and pistils may affect fruit development and seed setting rate.

### Effect of cgo-miR390s on fruits and seeds development in *Arabidopsis*

**Fig. 6:**
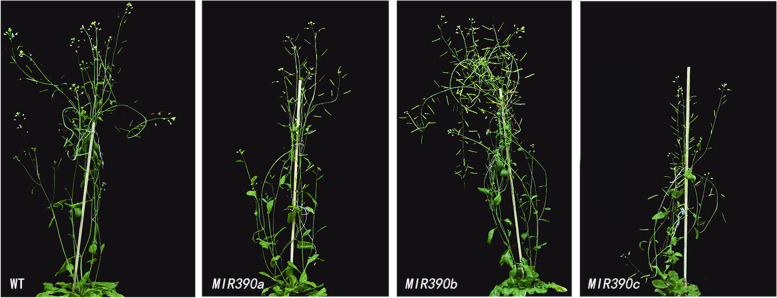
Fruit ripening observation of wild type and transgenic *Arabidopsis* plants

**Fig. 7:**
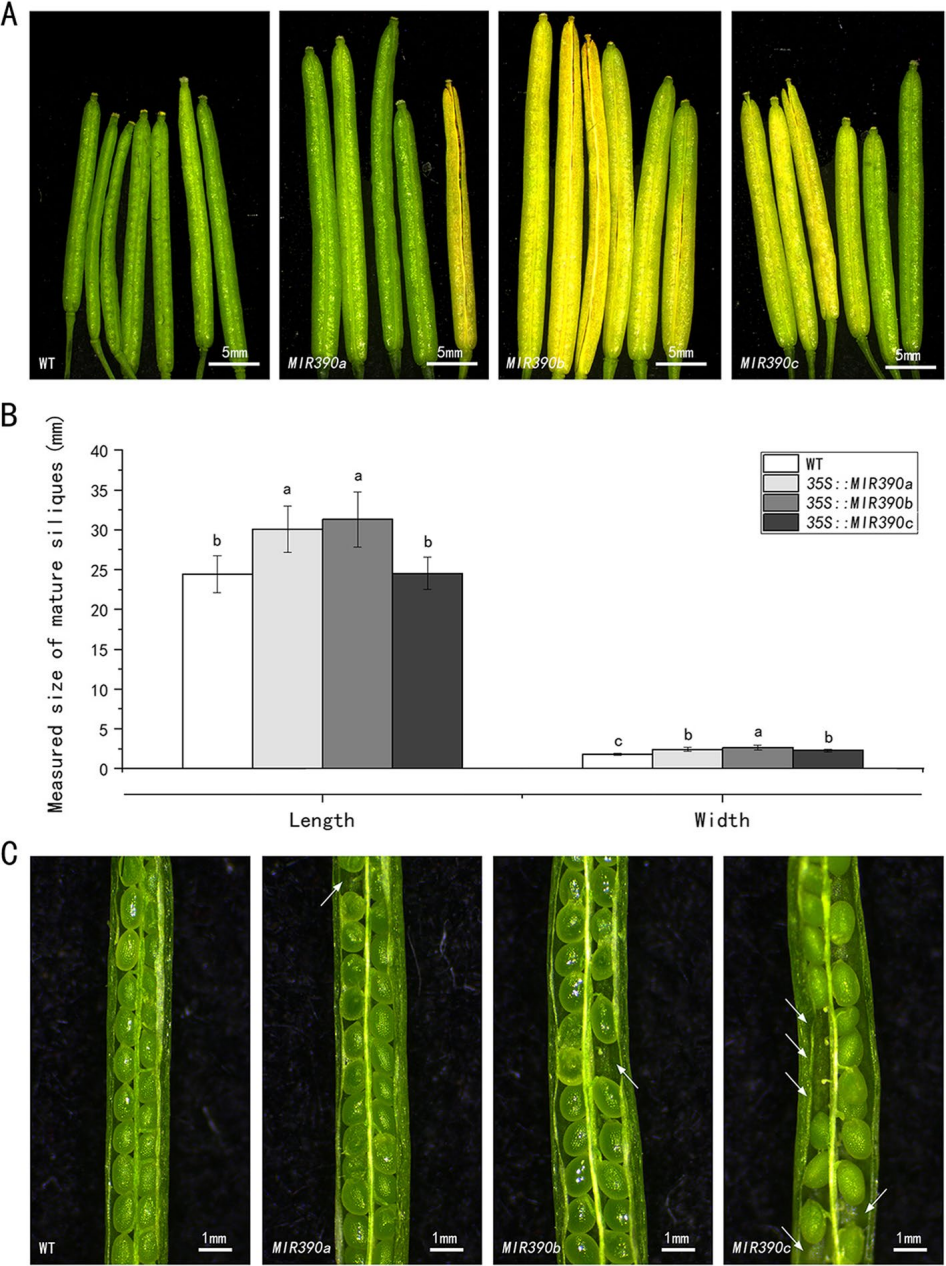
Phenotype changes in fruit pods of wild type and transgenic *Arabidopsis* plants. **A** Comparison of fruit morphology. **B** Measured size of mature siliques. The different letters indicate significant differences among 4 samples within a single group (*P* < 0.05). **C** Anatomical images of fruit pods. The white arrows point to absent or abort seeds

Although all transgenic plants flowered at a similar time as the wild-type, their senescence time was earlier than WT, especially in the reduced flowering (Fig. 6) and fruit ripening time (Fig. 7 A). By observing under a stereomicroscope, the fruit pods of all transgenic plants were thicker than those of the wild-type, with those of *cgo-MIR390a* and *MIR390b* overexpressed plants longer than *MIR390c* and WT (Fig. 7 A and B). The anatomical observation of fruits was further performed, which showed that the three genes overexpressed plants had different degrees of ovule abortion, and the seed volume of transgenic plants was larger than that of WT (Fig. 7 C). These results further indicated that cgo-miR390s were involved in fruit development by regulating the development of floral organs.

## Discussion

miR390s in plants are the ancient family of miRNAs with a high level of conservation among species [22]. Mature sequence analysis indicated that the three kinds of miR390s in *Cymbidium goeringii* were conserved. Secondary structure prediction showed that the mature miRNAs were all clustered in the same precursor. Studies of miRNA clusters in animals showed that evolutionarily conserved miRNAs are significantly enriched in clusters. The miRNAs in the same cluster have cooperative effects, which is called “functional co-adaptation” model [23]. We then analyzed the expression patterns by Quantitative real-time PCR (qRT-PCR), observing that the expression levels of cgo-miR390a-5p (miR390b-5p, miR390c-5p) and cgo-miR390a-3p.1 (miR390b-3p.1, miR390c-3p) were opposite in ovary but the expression trends of them in other floral organs were similar. During floral buds development, the highest expression of cgo-miR390a-5p and cgo-miR390a-3p.1 was obtained when the floral buds grew to 1-3 cm and significantly decreased after this stage. In particular, the expression pattern of miR390a-3p.2 (miR390b-3p.2) was different from the other two kinds of cgo-miR390s not only in floral organs but also in floral bud development. The above results indicated that there may be synergy between cgo-miR390a-5p and cgo-miR390a-3p.1, while miR390a-3p.2 were inclined to play roles independently. These inferences seemed to be further substantiated by the functional verification in *Arabidopsis*.

The overexpressed plants of cgo-miR390s cluster showed similar phenotypes in some of the organs, such as pod thickening, anther aspect ratio enlargement, and seed abortion. Moreover, the similarity of functions between *cgo-MIR390a* and *cgo-MIR390b* was more clear, while the phenotypes of *cgo-MIR390c*-overexpressed plants were different. This might be caused by the lack of a mature miRNA in *cgo-MIR390c*, which needs to be validated by further experiments. Of course, there are some differences between *cgo-MIR390a* and *cgo-MIR390b* as well, such as the effect on pods morphology. Although both *cgo-MIR390a* and *MIR390b* produce mature miRNAs, the stem-loop structure and precursor sequences between them were all different. In addition, the expression of mature miRNAs was not linearly associated with that of precursor miRNAs, which might be regulated at several levels [24]. These might be the reasons for their functional difference.

Next, how cgo-miR390s is involved in the regulation of reproductive organs development remains an open question, but we offer some possibilities. Existing studies indicated that miR390s play a potential role in the anther development and regulation of male-sterility in tomato, which was similar to the results in our study. The researchers speculated that it might be related to *TasiARFs*, even though their regulatory mechanisms are poorly understood [25]. In *Arabidopsis*, the miR390-*TAS3*-*ARF2/3/4* regulatory network is an integral part of the regulatory networks mediating auxin response [6, 26]. Flower phenotypes of *arf2* mutants showed infertility of the early-produced flowers, which was probably caused by the early elongation of gynoecium [27]. *TEX1*-and *TAS3*-mediated restriction of *ARF3* expression limits excessive megaspore mother cell formation [28]. The overexpressed plant of *cgo-MIR390c* seems to show a tendency towards these phenotypes. Moreover, the *ETT/ARF3* mutant exhibited some developmental abnormalities such as an expansion of the stylar and stigmatic regions [29]. Seed size and weight were dramatically increased in an *AtARF2* mutant [30]. These were very similar to all cgo-miR390s-overexpressed plants. Notably, three *cgo-MIR390s* overexpressed plants exhibited different degrees of ovule abortion, but Alexander staining and in vitro pollen germination assays showed that the pollen collected from transgenic *Arabidopsis* had normal viability. Therefore, we speculated that the ovule abortion of transgenic plants could result from aberrant development of female reproduction, such as stigma defect shown in Fig. 5 H, transmission tract defect and/or female gametophyte defects [31]. Of these, failed fertilization in *cgo-MIR390c*-overexpressed plants may also be caused by premature release of pollen grains. In addition, the miR390-*TAS3*-*ARF* pathway was also involved in other biological processes of non-model plants like the response to salt stress [32]. But this functionally important and archetypal regulatory pathway in land plants occurred significant variation [33]. All of these have brought more light to the verification experiments of target genes and the regulatory mechanism of cgo-miR390s in reproductive organs.

## Conclusions

In conclusion, cgo-miR390 family plays important roles in the reproductive organs of plants. Three kinds of cgo-miR390s displayed distinct temporal and spatial expression patterns during floral development in *Cymbidium goeringii*. The results also demonstrate that the overexpression of *MIR390s* alters the morphology and function of stamens and pistils in *Arabidopsis*, which affects the development of fruits and seeds. These findings preliminarily reveal the functions of cgo-miR390s and suggest that they can potentially be used for genetic improvement for orchid.

## Methods

### Plant materials and growth conditions

The Songmei cultivar of *C. cymbidium* was cultivated in a naturally lit glasshouse in the Institute of Horticulture, Zhejiang Academy of Agricultural Sciences (Hangzhou, Zhejiang, China). The *Arabidopsis* variety Columbia (Col-0) was used for gene overexpression and was grown at (22 ± 1) °C, 16 h day/8 h night in an artificial climate chamber.

### Extraction of RNA and qRT-PCR analysis

Total small RNA was extracted from the plants using MiniBEST Universal RNA Extraction Kit (Takara, Dalian, China). The total small RNA was then reverse transcribed into cDNA using miRNA 1st Strand cDNA Synthesis Kit (Vazyme, Nanjing, China) with stem-loop primer. qRT-PCR reaction solution was configured according to the instruction of ChamQ Universal SYBR qPCR Master Mix (Vazyme, Nanjing, China), and the action was performed using a StepOnePlus real-time PCR system (Thermofisher, Waltham, USA) under the following conditions: polymerase activation at 95℃ for 5 min, followed by 40 cycles at 95℃ for 15s and 60℃ for 1 min. We used 18 S rRNA and U6 as internal reference genes for *C. goeringii* and *Arabidopsis*, respectively. The threshold cycle (Ct) values of the PCR were averaged, and the relative transcript levels were quantified using the 2^−△△Ct^ method. The sequences of all the primers for this experiment are shown in Table 2.

**Table 2:** List of primers sequences used for the experiments

Sequence type	Gene name	Primer sequences
Precursor cloning	*cgo-MIR390a*	F: gagaacacgggggactctagaAAAAGCAGGGAGAAATCCTTAAAG
		R: ataagggactgaccacccgggGAGAGCAGGAAGAACCAATAAAACT
	*cgo-MIR390b*	F: gagaacacgggggactctagaACAAGTATGGGAGAACCATTAAAGC
		R: ataagggactgaccacccgggAAGAGCAAGAAGAACCCATAAAACTC
	*cgo-MIR390c*	F: gagaacacgggggactctagaAATGTATGGGAGAACCATTAAAGCT
		R: ataagggactgaccacccgggAGAGCAGGAAGAACCAATAGAACTCA
Reverse transcription	cgo-miR390a-5p	GTCGTATCCAGTGCAGGGTCCGAGGTATTCGCACTGGATACGACTGGCGC
	cgo-miR390a-3p.1	GTCGTATCCAGTGCAGGGTCCGAGGTATTCGCACTGGATACGACCTCAGG
	cgo-miR390a-3p.2	GTCGTATCCAGTGCAGGGTCCGAGGTATTCGCACTGGATACGACAAAACT
Fluorescence quantification	cgo-miR390a-5p	F: CGCGAAGCTCAGGAGGGATA
		R: AGTGCAGGGTCCGAGGTATT
	cgo-miR390a-3p.1	F: GCGCGTGTGACGCTATCTAT
		R: AGTGCAGGGTCCGAGGTATT
	cgo-miR390a-3p.2	F: CGCGCGGCTATCTATCCTG
		R: AGTGCAGGGTCCGAGGTATT
	18S	F: GGTCCTATTGTGTTGGCT
		R: TCGCAGTGGTTCGTCT
	U6	F: GGTGCTAAGAAGAGGAAGAAT
		R: CTCCTTCTTTCTGGTAAACGT

### Cloning of cgo-miR390 family and bioinformatic analysis

The putative precursors of cgo-miR390 family, *MIR390a* (145 bp), *MIR390b* (133 bp), and *MIR390c* (150 bp), were amplified with the primer pairs listed in Table 2, and then cloned into pBI121 vector at double restriction sites (*XbaI* and *SmaI*) to construct into recombinant vector. Gene amplification was performed by a standard PCR procedure, and the annealing temperatures were all 60 ℃.

Secondary structure and minimum free energy (MFE) prediction were performed with the online tool RNAfold (http://rna.tbi.univie.ac.at/). Homologous sequence of precursors and matures were download from miRbase (http://www.mirbase.org/index.shtml), and then aligned in DNAMAN 8.0 software. The phylogenetic tree was constructed using MEGA 6.0 with the neighbor joining (NJ) method.

### Transformation into Arabidopsis

pBI121-*35 S::MIR390a*, pBI121-*35 S::MIR390b* and pBI121-*35 S::MIR390c* were transformed into *Arabidopsis* Col-0 by the *Agrobacterium tumefaciens*-mediated floral dipping method [34]. T_0_ seeds were screened on MS media containing 50 mg L^− 1^ kanamycin to obtain T_1_ plants, and the gene insertions were confirmed by detection of genomic DNA PCR (Fig. S1). T_2_ plants were obtained through phenotypic observation and gene expression measurement. Screening was performed until homozygous lines of T_3_ generation were obtained.

### In vitro pollen germination and pollen-tube growth

Pollen was collected from fully open flowers and germinated on *Arabidopsis* thaliana pollen medium according to the procedure of Fan et al. [35] with minor modifications. This medium consisted of 1mM KCl, 10mM CaCl2, 0.8mM MgSO4, 1.5mM boric acid, 10 mg/L myo-inositol, 5mM Mes, 18% (w/v) sucrose and 1.0% (w/v) agarose. And the pH was adjusted to 5.8 using Tris-hydroxymethyl aminomethane (Tris) base. Pollen germination was determined by microscopy after 10 h incubation in a chamber at 25 ℃ and 100% relative humidity. The pollen grains were recorded as germinated when the pollen tube length was equal to or greater than the pollen grain diameter, as described by Boavida and McCormick [36].

### Alexander staining of stamens and pollen

For examination of stamen samples, the stamens were collected from flowers that were about to bloom, when the anthers had not yet split but the pollens were mature. The stamens were fixed in Alexander stain solution (Jisskang, Qingdao, China) at 55℃ for 3 h, and observed under the optical microscope.

For examination of pollen samples, 30 fully open flowers were randomly collected from the plants and mixed with ddH_2_O in a 1.5 mL centrifuge tube by vortex for 2 min, then took the flowers out. After centrifugation at 11,000 rpm for 1 min (at room temperature), the supernatant was discarded. The pollen precipitation was fixed in Alexander stain solution at 55℃ for 3 h. Then the morphology of pollen was observed under a microscope, and the number, coloration rate and aberration rate of pollen were counted.

### Scanning electron microscopy (SEM)

The pistils collected from fully open flowers were infiltrated in 4% glutaraldehyde (Electron Microscopy China, Beijing, China) overnight. Samples were dehydrated in an ethanol series, dried by EMITECH-K850 (Quorum, Hertfordshire, England) and sputter coated with Platinum. Images were taken with a Quanta 200 Scanning Electron Microscope (FEI, Hillsboro, USA) at an accelerating voltage of 25 kV.

## Supplementary Information

**Additional file 1.**

**Additional file 2.**

**Additional file 3.**

**Additional file 4.**

**Additional file 5.**

**Additional file 6.**

**Additional file 7.**

**Additional file 8.**

## Data Availability

All data generated or analyzed during this study are included in this published article and its supplementary information files. The precursor sequences and mature miRNAs of cgo-miR390s described here are available in the Supplemental Files and at Genbank: OM824433-OM824435.

## Abbreviations

miRNA: MicroRNA
Nt: nucleotide
ARF: Auxin response factor
ta: Trans-acting
AuxRE: Auxin-response element
MP: MONOPTEROS
LR: Lateral root
Se: Sepa
Li: Lip
La: Lateral petal
St: Stamen
Ov: Ovary
WT: Wild-type
qRT-PCR: Quantitative real-time PCR
Col: Columbia
