# Supplementary figures and images for: miR390 family of Cymbidium goeringii is involved in the development of reproductive organs in transgenic Arabidopsis
Source: BMC Plant Biol. 2022 Mar 26;22:149. doi: 10.1186/s12870-022-03539-3 (PMC8962573; doi:10.1186/s12870-022-03539-3)

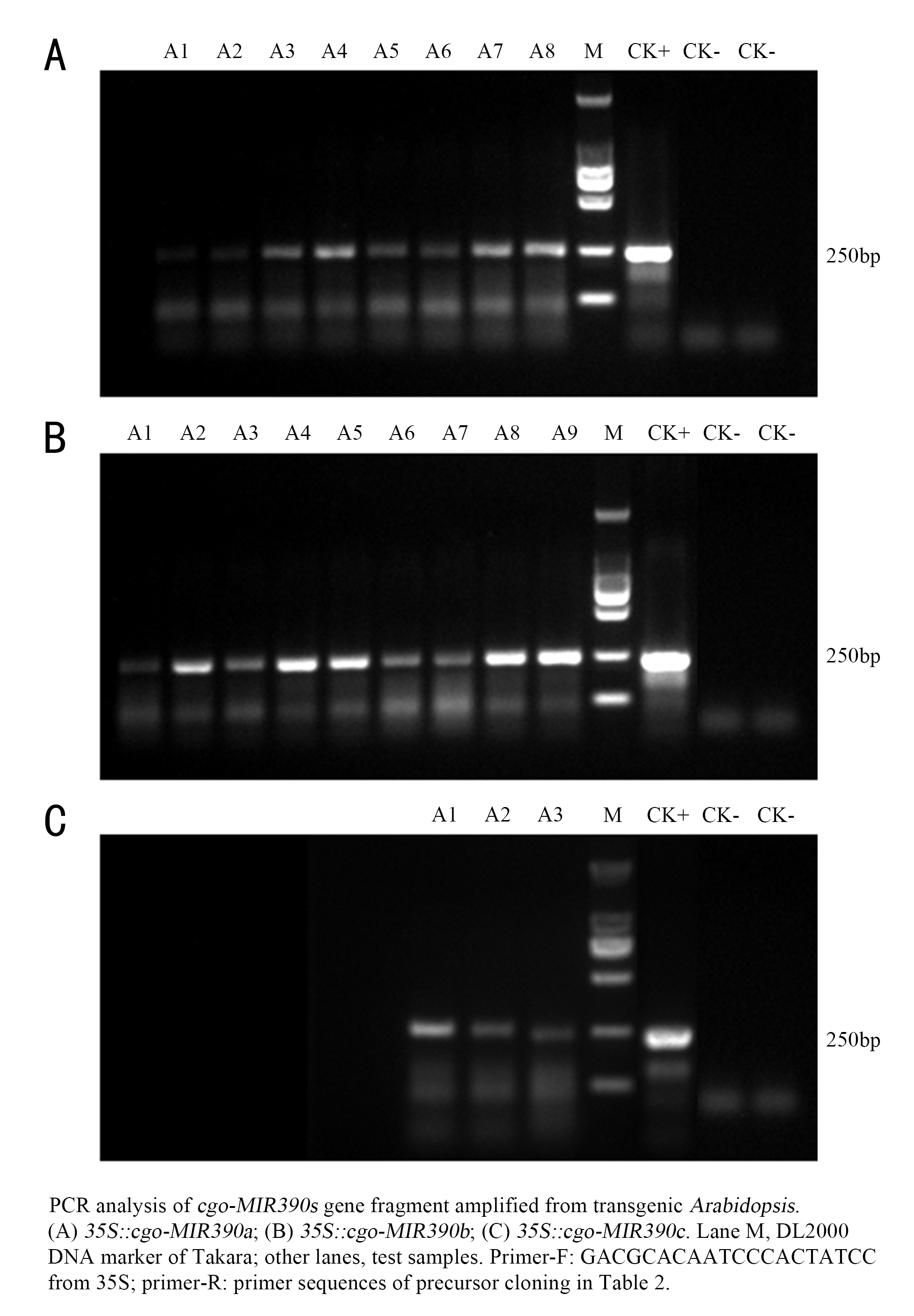

Supplement: Supplementary file 7 — Additional file 7. [file 12870_2022_3539_MOESM7_ESM.jpg]

Full-length gel of PCR analysis


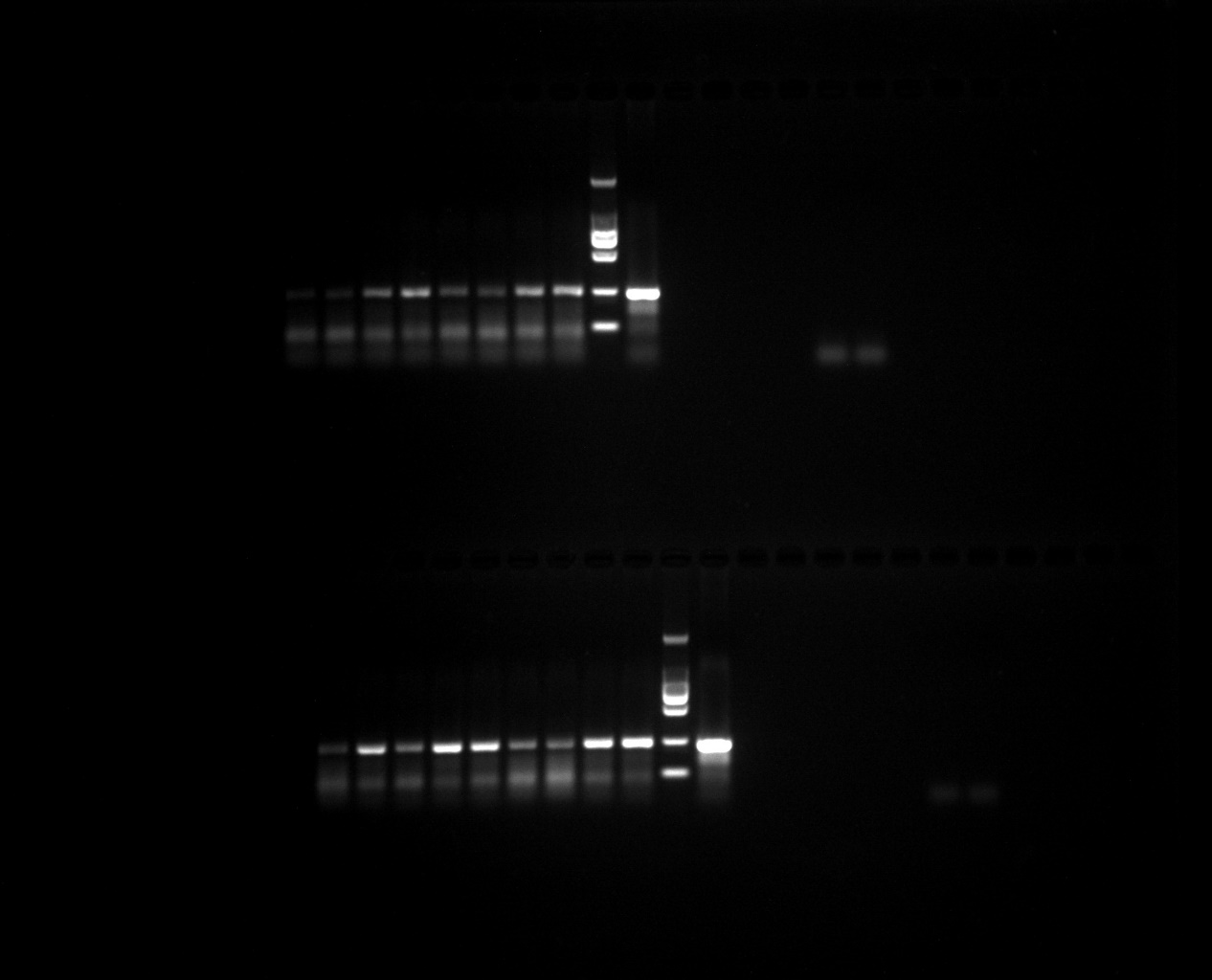


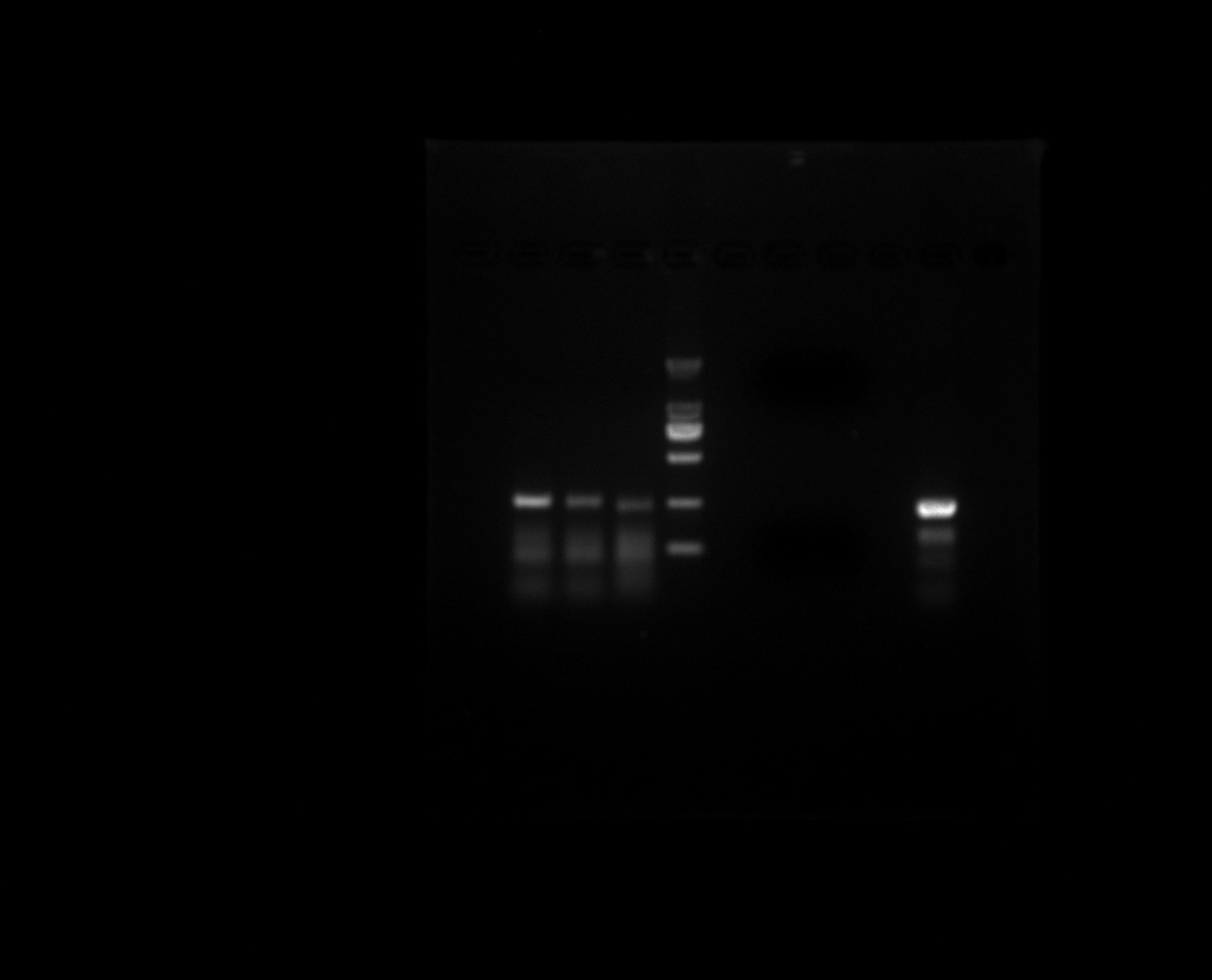

Supplement: Supplementary file 8 — Additional file 8. [file 12870_2022_3539_MOESM8_ESM.docx]
